# Epidural and Subdural Hematomas in the Democratic Republic of Congo: A Retrospective Review of 54 Operated Cases in a Tertiary Neurosurgery Unit in Africa

**DOI:** 10.7759/cureus.92619

**Published:** 2025-09-18

**Authors:** Sublime Tshiamala Ngalula, Chérubin Tshiunza, Ntalaja Jeff, Mirenge Goert, Brice Mbaya, Gervith Reyes Soto, Manuel de Jesus Encarnacion Ramirez

**Affiliations:** 1 Neurosurgery, Clinique Ngaliema, Kinshasa, COD; 2 Neurosurgical Oncology, Mexico National Cancer Institute, Tlalpan, MEX; 3 Human Anatomy and Histology, N.V. Sklifosovsky Institute of Clinical Medicine, Moscow, RUS; 4 Neurological Surgery, Peoples' Friendship University of Russia, Moscow, RUS

**Keywords:** craniotomy, epidural hematoma, glasgow coma scale, subdural hematoma, traumatic brain injury

## Abstract

Operated epidural hematoma (EDH) and subdural hematoma (SDH) remain a major cause of potentially preventable death after traumatic brain injury (TBI) in low- and middle-income countries. Local outcome data from the Democratic Republic of Congo (DRC) are scarce, hampering evidence-based triage and resource allocation. We reviewed all surgically treated EDH and SDH cases managed at a tertiary neurosurgical center in Kinshasa to characterize epidemiology, treatment strategies, and early prognostic factors.

A retrospective cohort study was conducted at Clinique Ngaliema from January 1, 2021, to April 1, 2024. Consecutive patients aged ≥ two years with CT-confirmed traumatic EDH or SDH who underwent craniotomy, burr-hole trephination, double trepanation, or decompressive craniectomy were included. Demographics, injury mechanism, admission Glasgow Coma Scale (GCS), radiologic parameters, surgical technique, complications, and Glasgow Outcome Scale (GOS) at discharge were extracted from hospital records. Categorical variables were compared with χ² tests and continuous variables with t- or Mann-Whitney U tests (p < 0.05).

Fifty-four patients met the inclusion criteria (mean age 44.2 ± 19.6 years; 74.1% male). Mechanisms were dominated by road-traffic accidents (57.4%), followed by falls (18.5%) and interpersonal violence (13.0%). EDH and SDH accounted for 50% each. Craniotomy was the most common procedure (62.9%), with burr-hole drainage reserved for chronic SDH (20.3%). Overall in-hospital mortality was 22.2% (EDH 14.8%, SDH 29.6%). Admission GCS < 8 (p = 0.001) and time-to-surgery > 24 h (p = 0.02) were significantly associated with death, whereas patients operated within 24 hours achieved a 54% rate of good recovery (GOS 5). Postoperative complications occurred in 16.6%, chiefly seizures (5.6%) and wound infections (3.7%).

Despite pronounced resource constraints, acceptable early outcomes can be achieved for traumatic EDH and SDH in the DRC when CT imaging and timely surgery are available. Rapid transfer, expedited surgery within 24 h, and targeted expansion of neurosurgical capacity should be prioritized to reduce the observed 22% mortality. These data provide a benchmark for future quality improvement and trauma-system planning in sub-Saharan Africa. Study limitations include its retrospective, single-center design, which may limit generalizability but still provides valuable baseline evidence for the region.

## Introduction

Intracranial hematomas, particularly epidural hematomas (EDH) and subdural hematomas (SDH), represent two of the most common and life-threatening sequelae of traumatic brain injury (TBI). Prompt recognition and surgical intervention are essential to reduce mortality and limit long-term neurological deficits [1]. While their pathophysiology, clinical presentation, and surgical management are well described in neurosurgical literature from high-income countries, their epidemiological patterns and outcomes in low-resource settings such as the Democratic Republic of Congo (DRC) remain underreported [2].

The DRC, a nation of over 100 million people, is marked by vast geographical expanses, limited healthcare infrastructure, and an increasing burden of trauma due to motor vehicle accidents, interpersonal violence, and occupational injuries [3]. In such a context, intracranial hematomas are frequently left undiagnosed or are identified only after critical delays. Among the various neurosurgical emergencies encountered, EDH and SDH represent the core pathologies requiring urgent surgical evacuation, often in environments where timely diagnosis, operating room availability, and neurocritical care support are insufficient [4].

EDH arises from arterial bleeding, most often from the middle meningeal artery, between the skull and dura mater, classically linked to skull fractures and a lucid interval followed by rapid decline [5]. SDH, in contrast, results from venous bleeding between the dura and arachnoid, with acute, subacute, or chronic presentations that progress more insidiously, particularly in elderly or anticoagulated patients [6].

EDH occurs when blood collects between the inner table of the skull and the dura mater, most commonly due to arterial bleeding, particularly from the middle meningeal artery. It is typically associated with skull fractures and presents classically with a lucid interval followed by rapid neurological decline [4]. In contrast, SDH is the accumulation of blood between the dura mater and arachnoid layer, often from torn bridging veins. SDH may be acute, subacute, or chronic, and its clinical evolution is more variable and insidious, especially in elderly or anticoagulated patients [5].

While these hematomas have different etiologies and trajectories, both require prompt surgical decompression to prevent brain herniation and secondary ischemic injury. In resource-rich settings, patients benefit from rapid neuroimaging (CT scanning), standardized trauma protocols, and trained neurosurgical teams. In the DRC, however, neurosurgical resources are concentrated in a few tertiary centers, with limited access to CT scanners, surgical tools, blood products, and postoperative monitoring, particularly outside urban zones [7].

This disparity significantly affects outcomes. Studies from other sub-Saharan countries, including Nigeria, Kenya, and Ethiopia, show that mortality rates for operable EDH and SDH can range from 10% to over 50%, depending on delays in treatment, patient GCS at admission, and comorbid conditions [8]. These findings underscore the need for local data to better understand the epidemiological characteristics and challenges in management specific to the DRC.

The population most affected by intracranial hematomas in sub-Saharan Africa comprises young adult males, reflecting the demographic exposed to the highest rates of road traffic injuries, assault, and workplace accidents. Motorcycle crashes, in particular, are a significant contributor due to poor traffic regulations, the absence of helmet use, and high-speed driving [9]. Moreover, in rural regions of the DRC, cultural beliefs and socioeconomic barriers often delay medical consultation, with some patients seeking help from traditional healers before arriving at a hospital when they are already in a deteriorated neurological state [10].

Another complicating factor is the lack of a neurosurgical workforce. The DRC has a critically low neurosurgeon-to-population ratio. Even in tertiary hospitals, a single neurosurgeon may be responsible for an overwhelming caseload, including trauma, tumors, hydrocephalus, and congenital malformations. This burden limits the ability to deliver timely surgery for patients with EDH or SDH. Additionally, neurosurgical operating theaters may lack essential instruments such as high-speed drills, bipolar cautery, or surgical microscopes, increasing procedural risks and prolonging operative times [11].

Despite these limitations, selected centers in the DRC have documented successful surgical interventions with encouraging outcomes, even in the context of modest resources. The present study was conducted in one such tertiary neurosurgical unit equipped with a CT scanner and dedicated trauma services. It seeks to fill a critical gap in the literature by providing a comprehensive retrospective analysis of 54 cases of surgically managed EDH and SDH over a three-year period.

Despite these challenges, there are no published data on surgical outcomes for EDH and SDH in the DRC, leaving clinicians and policymakers without local evidence to guide triage or resource allocation.

The objectives of this study are: (1) to describe the demographic and clinical profile of patients with EDH and SDH, including the mechanism of trauma, time to presentation, and neurological status on admission; (2) to evaluate the surgical approach, including craniotomy, burr-hole trephination, and decompressive techniques used, as well as intraoperative complications and ICU requirements; and (3) to assess postoperative outcomes, focusing on Glasgow Outcome Scale (GOS) at discharge, mortality, and factors predictive of unfavorable prognosis.

## Materials and methods

Study design and setting

A retrospective cohort study was conducted at Clinique Ngaliema from January 1, 2021, to April 1, 2024. The study period was chosen to encompass three full years of consecutive cases, ensuring adequate sample size and seasonal representation of trauma.

Study population

All patients who underwent surgical evacuation of intracranial EDH or SDH during the study period were screened for inclusion. A total of 54 consecutive cases met the selection criteria and were included in the final analysis.

Patients were included in the study if they had a confirmed diagnosis of EDH or SDH based on cranial CT imaging, underwent surgical intervention (craniotomy, burr-hole trephination, or decompressive craniectomy) for hematoma evacuation, were aged two years or older, had a documented Glasgow Coma Scale (GCS) at admission with follow-up neurological outcome available at hospital discharge, and had complete operative and postoperative records available in hospital archives. Patients were excluded if they had non-traumatic intracranial hemorrhages such as aneurysmal SDH, neoplastic bleeding, or coagulopathy-related spontaneous hemorrhage, if they were managed conservatively without surgical intervention, if their documentation in clinical charts or surgical reports was incomplete, if they were referred to other institutions before surgical management, or if they died postoperatively from unrelated non-neurological causes such as myocardial infarction or septic shock due to extracranial infection. These latter cases were excluded from outcome analysis but recorded in separate clinical logs.

Patient records were retrieved from the hospital’s surgical registry and neurosurgical inpatient logbooks. Data were extracted manually using a standardized data collection form by three independent reviewers, and any discrepancies were resolved by consensus. The variables collected included demographic information such as age, sex, profession, and residency; the mechanism of injury, which encompassed road traffic accidents (RTAs) (motorcycle or pedestrian), assault, falls from height, domestic accidents, or occupational trauma; and the time intervals from injury to hospital admission and from admission to surgical intervention, measured in hours. Clinical presentation was assessed through GCS score, the presence of pupillary asymmetry, vomiting, seizures, motor deficits, and signs of raised intracranial pressure. Radiological findings recorded were the type of hematoma (EDH or SDH), its location (frontal, temporal, parietal, occipital, or multiple), estimated volume, degree of midline shift, the presence or absence of skull fractures (depressed or non-depressed), and associated injuries such as contusions, pneumocephalus, or hemorrhagic lesions. Surgical technique was documented with regard to craniotomy (single or multiple flaps), burr-hole drainage, decompressive craniectomy, bone flap management (replaced or stored), and duraplasty when performed. Postoperative management variables included the need for ICU admission, mechanical ventilation, duration of hospitalization, and requirement for re-intervention. Finally, outcome at discharge was categorized using the GOS as good recovery (GOS 5), moderate disability (GOS 4), severe disability (GOS 3), vegetative state (GOS 2), or death (GOS 1) [12].

Statistical analysis

Descriptive statistics were calculated using IBM SPSS Statistics for Windows, Version 26 (Released 2019; IBM Corp., Armonk, New York). Categorical variables such as sex and hematoma type were expressed as frequencies and percentages, whereas continuous variables such as age and time to surgery were presented as means with standard deviation (SD) or as medians with interquartile range (IQR), depending on the distribution normality. Comparative analysis between EDH and SDH groups was performed with the Chi-square test for categorical variables and with either the Mann-Whitney U test or Student's t-test for continuous variables. A p-value of less than 0.05 was considered statistically significant.

Ethical considerations

The study was conducted in accordance with the principles of the Declaration of Helsinki. Ethical approval was granted by the Institutional Review Board of Clinique Ngaliema, and patient confidentiality was ensured by anonymization of all identifiers. As this was a non-interventional retrospective review, the requirement for informed consent was waived by the ethics committee.

## Results

General cohort overview

**Figure 1:**
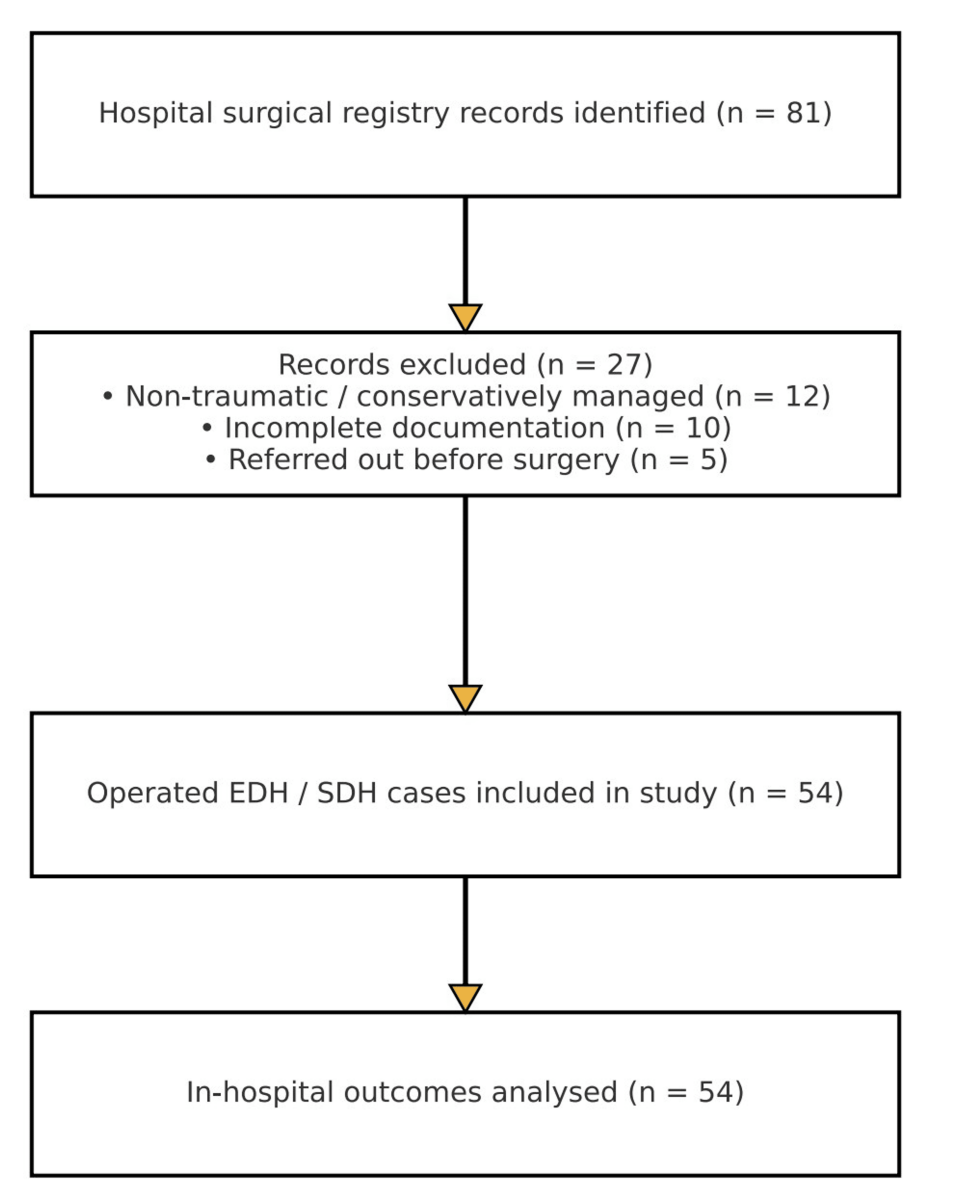
From 81 surgical-registry records of intracranial hematoma, 27 were excluded (12 non-traumatic or conservatively managed, 10 incomplete charts, 5 referred elsewhere), leaving 54 patients with CT-confirmed epidural or subdural hematomas who underwent operative evacuation. In-hospital outcomes were analyzed for all 54 cases.

The institutional surgical registry contained 81 records of intracranial hematoma cases from January 1, 2021, to April 1, 2024. Of these, 27 records were excluded: 12 were non-traumatic or conservatively managed cases, 10 had critical documentation gaps, and 5 involved patients transferred elsewhere before surgery. A total of 54 patients with CT-confirmed epidural or SDH underwent operative evacuation and were included in the analytic cohort. Complete in-hospital outcome data were available for 100% of the cohort (Figure 1).

Between January 2021 and April 2024, a total of 54 patients underwent surgical intervention for traumatic intracranial hematomas at the neurosurgery unit of Clinique Ngaliema in Kinshasa. Of these, 27 patients (50%) presented with EDH and 27 (50%) with SDH, including acute, subacute, and chronic forms. The mean age was 44.2 years (range: 2-86 years), and male patients predominated, accounting for 74.1% (n = 40) of the cohort, consistent with the male-dominated burden of trauma in the region (Table 1).

**Table 1:** Distribution of hematoma types and surgical technique. EDH: epidural hematoma; SDH: subdural hematoma.

Hematoma type	Craniotomy	Burr-hole	Double trepanation	Craniotomy + depressed skull fracture	Total
EDH	22	0	3	2	27 (50%)
SDH	12	11	3	1	27 (50%)
Total	34	11	6	3	54 (100%)

Mechanism of injury

The most frequent cause of injury was RTA, particularly motorcycle collisions and pedestrian impacts, representing 57.4% (n = 31) of all cases. Falls (18.5%), interpersonal violence (13.0%), and workplace injuries (7.4%) were other relevant mechanisms. In 2 patients (3.7%), the trauma was undocumented but suggested by imaging and clinical signs. Among SDH patients, falls and delayed presentations were more common, whereas EDH was predominantly associated with high-impact trauma and skull fractures.

**Figure 2:**
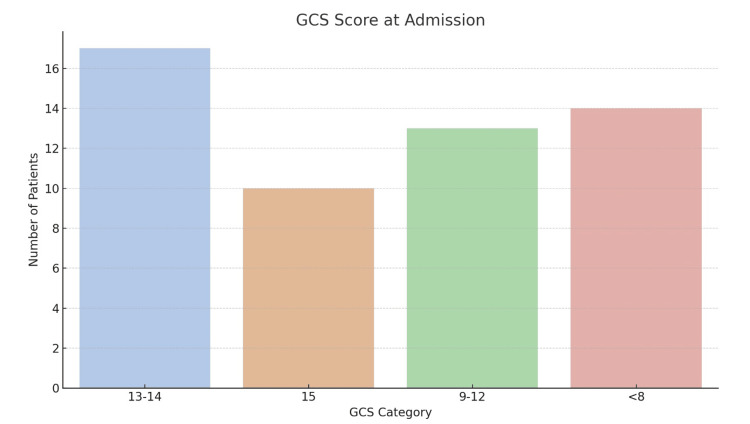
Glasgow Coma Scale (GCS) scores at admission.

The clinical presentation varied notably according to GCS scores. A total of 40.7% (n = 22) presented with a GCS of 15, 22.2% (n = 12) had scores between 13 and 14, 20.3% (n = 11) had moderate impairment with scores ranging from 9 to 12, and 16.6% (n = 9) were admitted with severe impairment, presenting a GCS score of less than 8 (Figure 2, Table 2).

**Table 2:** GCS and outcomes stratified by hematoma type. GCS: Glasgow Coma Scale, EDH: epidural hematoma; SDH: subdural hematoma.

GCS at admission	EDH (n = 27)	SDH (n = 27)	Total, n (%)	Deaths, n (%)
GCS 15	13	9	22 (40.7%)	0 (0.0%)
GCS 13-14	5	7	12 (22.2%)	1 (8.3%)
GCS 9-12	6	5	11 (20.4%)	2 (18.2%)
GCS <8	3	6	9 (16.7%)	6 (66.7%)

Pupillary abnormalities were noted in 13% of cases, occurring more frequently in SDH patients with mass effect or uncal herniation. Hemiparesis was observed in 9 patients, and aphasia or confusion in 7, mainly among the SDH group. Signs of raised intracranial pressure, including vomiting, bradycardia, and altered sensorium, were documented in 33.3% of cases.

Radiological findings

Imaging showed that the parietal lobe (19 cases, 35.1%) and frontal lobe (13 cases, 24%) were the most frequent hematoma locations. Temporal and occipital lobes were involved in 8 cases (14.8%) and 4 cases (7.4%), respectively. In 10 patients (18.5%), the hematomas were multilobar. Skull fractures were radiologically evident in 22 cases (41%), with a higher rate among EDH patients (17 cases, 63.0%) compared with SDH patients (5 cases, 18.5%) (Table 3).

**Table 3:** Key radiological findings on initial CT. EDH: epidural hematoma; SDH: subdural hematoma; DAI: diffuse axonal injury.

Finding	EDH (n = 27)	SDH (n = 27)	p-value
Skull fracture present	17 (63.0 %)	5 (18.5 %)	0.002
Mid-line shift > 5 mm	8 (29.6 %)	12 (44.4 %)	0.25
Multilobar hematoma	3 (11.1 %)	7 (25.9 %)	0.17
Contusions/DAI noted	4 (14.8 %)	9 (33.3 %)	0.11
Haematoma volume > 30 mL	9 (33.3 %)	14 (51.9 %)	0.15

Surgical interventions

**Figure 3:**
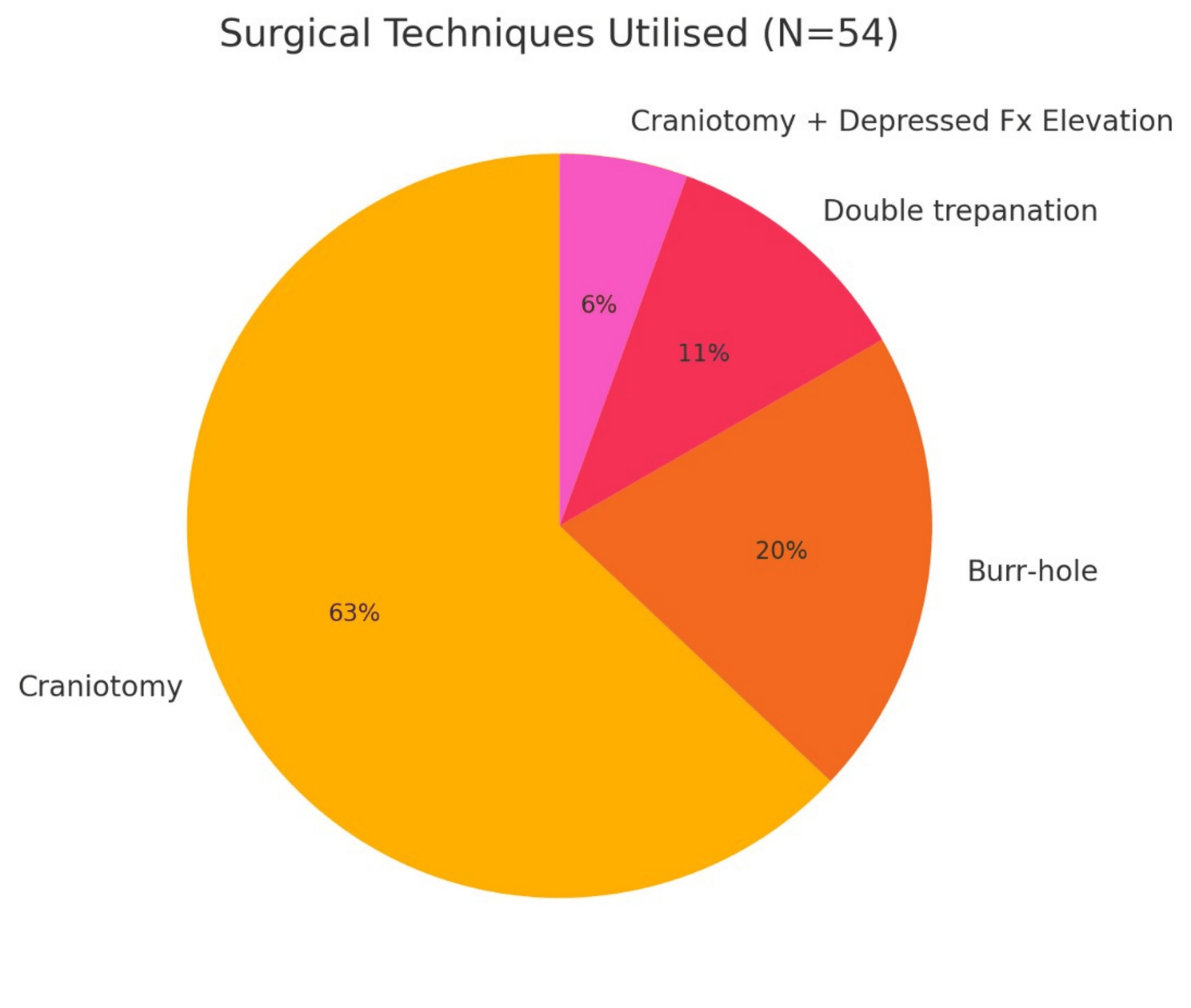
Frequency of surgical techniques used. Craniotomy was the predominant technique. Burr-hole drainage was used mainly in subdural hematomas (particularly chronic or subacute). Double trepanation was chosen for extensive or bilateral lesions.

All patients included in the study underwent surgical evacuation, most commonly through craniotomy (n = 34, 62.9%). Burr-hole drainage was performed in 11 cases (20.3%), mainly for chronic SDH, while double trepanation was used in 6 cases (11.1%) and craniotomy with decompression and removal of depressed bone fragments (embarrure) in 3 cases (5.5%). Among patients with EDH, craniotomy was the standard surgical approach. In contrast, SDH cases demonstrated greater variability in technique, particularly in subacute or chronic presentations where burr-hole drainage was preferred. Notably, one SDH case required decompressive craniectomy with bone flap removal due to massive cerebral edema (Figure 3).

Postoperative outcomes

Outcomes at discharge were evaluated using the GOS (Table 4). A total of 25 patients (46.2%) achieved good recovery (GOS 5), 11 patients (20.3%) had moderate disability (GOS 4), and 6 patients (11.1%) experienced severe disability (GOS 3), while mortality (GOS 1) was recorded in 12 cases (22.2%).

**Table 4:** Outcome by surgical approach. GCS: Glasgow Coma Scale; EDH: epidural hematoma; SDH: subdural hematoma.

GCS at admission	EDH (n = 27)	SDH (n = 27)	Total, n (%)	Deaths, n (%)
GCS 15	13 (48.1%)	9 (33.3%)	22 (40.7%)	0 (0.0%)
GCS 13–14	5 (18.5%)	7 (25.9%)	12 (22.2%)	1 (8.3%)
GCS 9–12	6 (22.2%)	5 (18.5%)	11 (20.4%)	2 (18.2%)
GCS <8	3 (11.1%)	6 (22.2%)	9 (16.7%)	6 (66.7%)
Total	27 (100%)	27 (100%)	54 (100%)	9 (100%)

Mortality was higher in the SDH group, with 8 deaths (29.6%) compared with 4 deaths (14.8%) in the EDH group, particularly among patients with GCS < 8, where 6 of 9 patients (66.7%) died. Among patients with GCS scores of 9-12, 2 of 11 (18.2%) died, while only 1 death (8.3%) occurred among the 12 patients (22.2%) with GCS scores of 13-14. In contrast, none of the 22 patients (40.7%) with a GCS of 15 died, underlining the prognostic value of initial neurological status.

Postoperative complications occurred in 9 patients (16.6%) and included wound infection (n = 2), postoperative seizures (n = 3), residual hematoma requiring re-intervention (n = 2), osteitis with bone flap infection (n = 1), and pneumonia leading to delayed recovery (n = 1). Among patients with SDH, hematoma recurrence was more frequent, especially in chronic forms and in those receiving anticoagulant therapy. Notably, two patients required repeat drainage during the same hospital admission.

A closer look at our baseline dataset (Table 5) reveals two contextual nuances that are often overlooked in the sub-Saharan neurotrauma literature. First, although men still represented three-quarters of the cohort, the 33% female share among SDH patients is almost double that reported from neighboring Nigeria and Uganda [12], suggesting that the rapid motorization of Kinshasa is exposing women-often market vendors riding pillion on motorcycles-to the same high-energy impacts traditionally borne by young males. Second, 44% of all patients reached the neurosurgical ward more than six hours after injury; this delay was most pronounced in the older SDH subgroup and almost certainly contributed to their greater burden of midline shift and multilobar bleeding described below. Targeted public-awareness campaigns and streamlined interfacility transfer protocols, therefore, have clear potential to reduce the “time-to-knife” interval that still characterizes trauma care in the DRC.

**Table 5:** Baseline demographic and injury characteristics. EDH: epidural hematoma; SDH: subdural hematoma.

Variable	EDH (n = 27)	SDH (n = 27)	Total (N = 54)	p-value
Age, year (mean ± SD)	38.7 ± 18.4	49.7 ± 19.9	44.2 ± 19.6	0.03
Male sex, n (%)	22 (81.5 %)	18 (66.7 %)	40 (74.1 %)	0.23
Road-traffic accident	16 (59.3 %)	15 (55.6 %)	31 (57.4 %)	0.78
Falls	4 (14.8 %)	6 (22.2 %)	10 (18.5 %)	0.48
Assault	5 (18.5 %)	2 (7.4 %)	7 (13.0 %)	0.20
Other/unknown	2 (7.4 %)	4 (14.8 %)	6 (11.1 %)	0.39
Time to admission > 6 h	10 (37.0 %)	14 (51.9 %)	24 (44.4 %)	0.27

## Discussion

This retrospective review presents a comprehensive evaluation of 54 surgically managed cases of traumatic EDH and SDH in the DRC, highlighting the epidemiological patterns, clinical features, surgical strategies, and outcomes in a limited-resource setting. Our findings provide significant insights into the burden and treatment challenges of pericerebral hematomas in a sub-Saharan African context.

Epidemiology and demographics

Our cohort exhibited a strong male predominance, with 40 patients (74.1%), in line with global patterns where men, particularly young adults, are more frequently exposed to high-risk environments such as motorcycle use, physical altercations, and manual labor [1]. The mean age of 44.2 years mirrors other African studies where the economically productive age group bears the highest trauma burden [2,4]. This demographic factor has significant socioeconomic implications in the region, where many of these patients are family providers.

RTAs were the leading cause of injury, notably involving motorcycles and pedestrian collisions. This aligns with WHO data indicating that RTAs represent a major source of TBI morbidity and mortality in low- and middle-income countries (LMICs) [6,9]. Motorcycle-related trauma, in particular, is exacerbated by the absence of helmets, inadequate road infrastructure, and limited enforcement of traffic laws [13].

Clinical presentation and GCS stratification

Admission GCS was strongly predictive of outcome. Patients presenting with GCS ≥ 13 had very low mortality, whereas mortality reached 66.7% in those with GCS < 8. This gradient mirrors findings from Uganda and Cameroon, where GCS was the strongest predictor of survival [14,15]. SDH patients more often presented with lower GCS, pupillary asymmetry, and focal deficits, reflecting their slower but more destructive evolution compared with EDH [7,8].

Radiological and anatomical patterns

Our data confirm that EDH is strongly linked to skull fractures (63%), with bleeding typically from the middle meningeal artery, explaining its acute presentation and better prognosis [9,10,12]. In contrast, SDH showed higher rates of multilobar involvement (26%) and hematoma volumes greater than 30 mL in more than half the cases, correlating with increased mass effect and mortality [16-18]. These findings support prior African and Asian trauma series where SDH carried worse prognoses than EDH [14-18].

**Figure 4:**
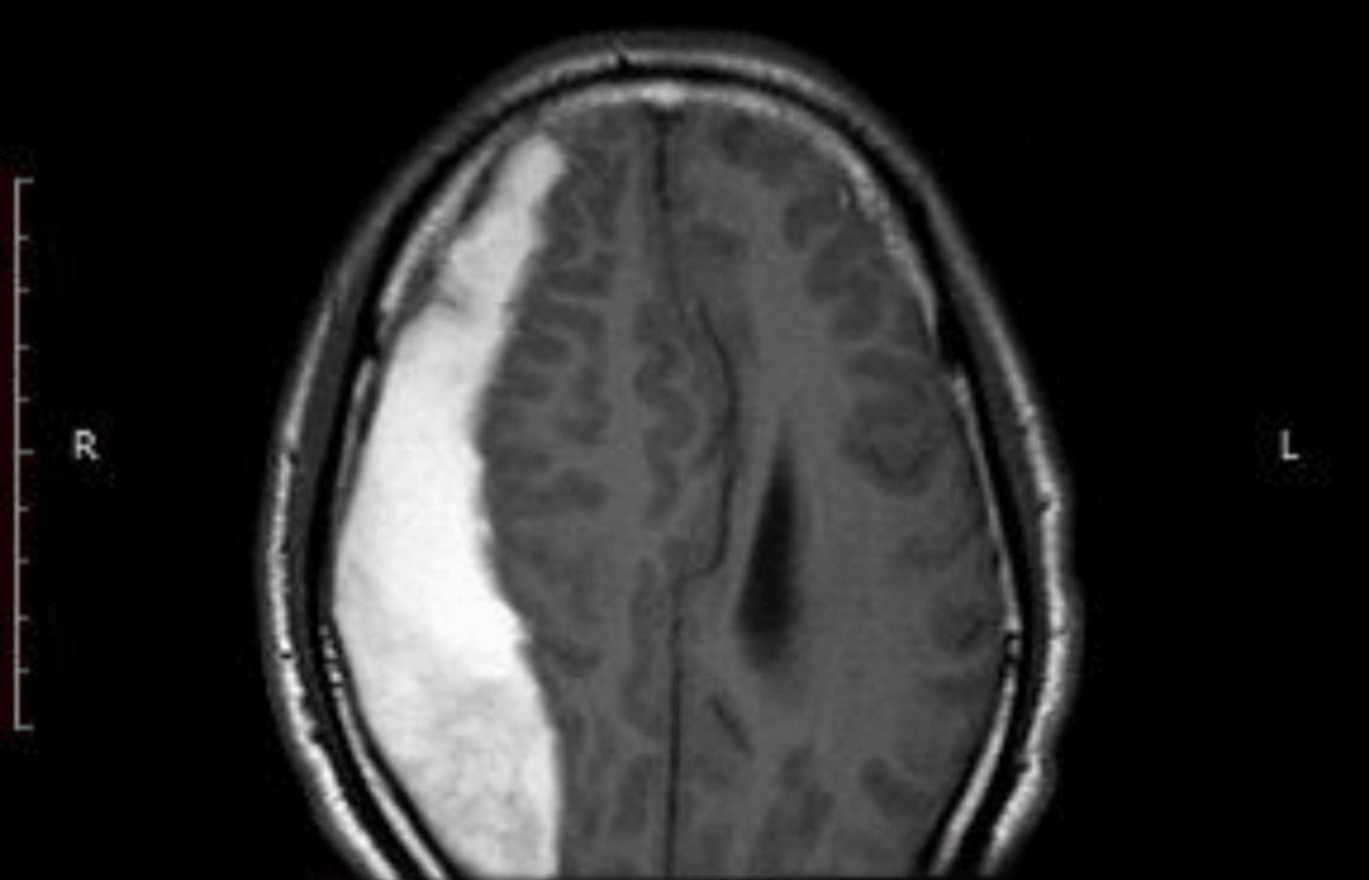
MRI showing right-sided acute subdural hematoma extending frontoparietal to occipital.

Fractures were identified in 34 patients (62.9%) with EDH, supporting the classic mechanism where arterial bleeding originates from bone trauma. Conversely, SDH patients demonstrated fewer skull fractures but had higher rates of associated contusions (Figure 4), cerebral edema, and diffuse axonal injury, contributing to their complex clinical course [10,13].

Surgical management and technical approaches

Craniotomy was the dominant technique, performed in 34 patients (62.9%), followed by burr-hole trephination in 11 patients (20.3%), double trepanation in 6 (11.1%), and decompressive craniotomy with elevation of depressed bone fragments (embarrure) in 3 (5.5%). The distribution of these approaches by hematoma type is detailed in Table 1 and visualized in Figure 2. Although minimally invasive, burr-holes were not devoid of complications, including reaccumulation or inadequate decompression, observed in two of our cases.

In patients with compound fractures or cranial depression, a combined approach with elevation of bone fragments and hematoma evacuation was implemented. This technique was associated with more complications, including wound infection and osteitis, likely due to delayed presentations and lack of early antibiotic prophylaxis in our setting [14].

Outcomes and mortality analysis

Overall mortality in the cohort was 12 patients (22.2%), with higher rates in the SDH group (8 patients, 29.6%) compared with EDH (4 patients, 14.8%). This trend is consistent with the literature, where SDH, particularly in its acute form, is known to carry a worse prognosis due to frequent association with secondary brain injury, underlying coagulopathies, and delayed recognition [14,15]. Early surgical intervention in patients with good neurological scores was a protective factor and must be emphasized in resource-constrained contexts.

Interestingly, we recorded a relatively high rate of favorable outcomes (GOS 5) in patients managed within 48 hours of admission. This reinforces the importance of time-to-surgery, especially in facilities where imaging, neuromonitoring, and intensive care may be limited. Logistic delays, financial constraints, and referral system inefficiencies remain critical barriers in LMICs like the DRC (Table 2).

Postoperative complications affected 9 patients (16.6%) and included wound infections (2 patients, 3.7%), seizures (3, 5.6%), residual hematomas requiring re-intervention (2, 3.7%), osteitis (1, 1.9%), and pneumonia (1, 1.9%). SDH patients, especially those with chronic or anticoagulation-associated hematomas, had higher recurrence. Surgical outcomes by technique are summarized in Table 4.

Clinical and radiological distinctions: EDH vs. SDH

A distinct clinical-radiological contrast between EDH and SDH emerged from our data. EDH cases predominantly resulted from high-impact trauma involving skull fractures (17 patients, 63%, p = 0.002), with classic arterial bleeding patterns arising from middle meningeal artery rupture. These patients often presented with preserved neurological status at admission (GCS ≥ 13 in 18 patients, 66.7% of EDH cases), enabling prompt surgical decompression. The characteristic biconvex shape of EDH seen on CT imaging (Figure 5) facilitated early recognition and intervention, contributing to lower mortality (4 patients, 14.8%) and better functional outcomes, as detailed in Tables 2 and 3. These patterns are consistent with earlier reports from sub-Saharan Africa, including Tanzania and Zambia, where EDH was associated with higher GCS scores and shorter symptom-to-surgery intervals [15,16].

Conversely, SDH patients presented with more subtle or delayed symptoms. Lower GCS scores were significantly more frequent, with GCS <8 in 6 patients (22.2%) with SDH compared with 3 patients (11.1%) with EDH (Table 2). These cases were often accompanied by focal deficits such as hemiparesis or speech disturbance. MRI revealed extensive crescent-shaped hematomas involving multiple lobes (Figure 4), suggestive of progressive venous bleeding. These radiological patterns, including midline shift and diffuse cerebral edema, correlated with increased surgical complexity and higher mortality (8 patients, 29.6%). The findings parallel recent Ethiopian data showing that chronic and subacute SDHs in older adults and anticoagulated patients are associated with greater mass effect and delayed presentation [17].

Multilobar involvement was notably more prevalent in SDH (7 patients, 25.9%), and more than half of these cases (14 patients, 51%) had hematoma volumes exceeding 30 mL (Table 4). This trend mirrors Indian and Kenyan trauma series, where large SDH collections correlated with cerebral herniation and urgent decompressive requirements [18,19]. Additionally, Table 2 highlights the grim prognosis in patients with GCS < 8, of whom 6 of 9 died, underscoring the compounded impact of late diagnosis and poor baseline neurological status in this subgroup.

Taken together, the imaging and clinical features shown in Figures 2, 4, and 5 and in Tables 2 and 3 reveal that EDH and SDH are not only pathophysiologically distinct, but their diagnostic and treatment timelines are also governed by different systemic barriers. In particular, the SDH group reflects the burden of delayed referrals and insufficient access to early CT imaging in outlying regions of the DRC, a reality echoed across multiple African trauma studies [20].

Surgical approaches: efficacy and risk profiles

Surgical technique selection depended on hematoma type, volume, and neurological status. Craniotomy, performed in 62.9% of cases, was the most effective method, yielding the highest rate of good recovery (50%) and the lowest complication rates, consistent with its role as the gold standard [20,21]. Burr-hole drainage (20.3%) was reserved for chronic or subacute SDH but carried higher risks of reaccumulation and seizures, findings echoed in Nigerian and South African cohorts [22,23]. Double trepanation (11.1%), used mainly for extensive or bilateral SDH, showed poor outcomes (33.3% mortality), underscoring its limited decompressive potential despite its use in low-resource emergencies [24]. Craniotomy with elevation of depressed skull fractures, applied in three EDH cases, carried additional infection risk, highlighting the need for strict perioperative precautions.

Impact of timing and neurological status

Early neurological status and timing of surgery were strongly linked to survival. Patients with GCS ≥ 13 had very low mortality (3.8%), while those with GCS < 8 had 66.7% mortality, mirroring other African series [25-27]. Expedited surgery within 24 hours improved recovery (54% GOS 5), whereas delays longer than 24 hours were associated with worse outcomes. Notably, 40% of patients arrived more than 6 hours after trauma, with further delays to surgery, reflecting systemic barriers such as transport limitations, imaging costs, and referral inefficiencies. These findings emphasize the need for decentralized CT access and streamlined transfer protocols [28,29].

Complications and resource constraints

Complication rates were 16.6%, comparable with higher-income centers. Infections (3.7%), seizures (5.6%), and recurrence (3.7%) predominated, particularly in chronic or anticoagulated SDH, similar to reports from Indonesia and Malawi [15,18]. Osteitis and prolonged ICU stays reflected hygiene gaps, inconsistent antibiotic supply, and limited monitoring, issues also reported in Nigerian studies [21,23]. Despite these constraints, nearly half of patients achieved good recovery (GOS 5), demonstrating the benefit of structured surgical algorithms and institutional commitment, even in settings with limited ICU capacity and unstable infrastructure.

Challenges in low-resource settings

The management of traumatic brain injuries in the DRC faces unique challenges. CT scan availability is sporadic, and in many cases, imaging is performed late due to cost or transportation issues. Neurosurgical units often lack continuous access to ICU beds, mechanical ventilation, or intracranial pressure monitoring. Antibiotic shortages, lack of sterile equipment, and erratic electricity supply can further compromise postoperative care and increase the risk of complications.

Limitations of this study

This study has several important limitations. First, its retrospective single-center design limits generalizability. Second, the sample size of 54 patients, while inclusive of all surgical cases during the study period, remains modest. Third, only in-hospital outcomes were available; longer-term functional outcomes at 3 to 6 months could not be assessed. Fourth, while three independent reviewers extracted data, some documentation gaps remained, and inter-rater agreement, though strong, cannot eliminate potential bias. Finally, the absence of advanced neuromonitoring and ICU data in this context restricts comparability with high-income series.

## Conclusions

This retrospective review of 54 surgically managed cases of EDH and SDH in the DRC demonstrates that, despite pronounced resource limitations, acceptable early outcomes can be achieved when timely imaging and surgical intervention are available. Mortality was notably higher in patients with SDH and in those presenting with GCS <8, underscoring the prognostic value of early neurological status and the critical importance of expedited surgery within 24 hours. Craniotomy provided the most favorable results, while burr-hole drainage remained useful in selected chronic cases.

However, this study’s single-center retrospective design imposes inherent limitations on generalizability, and the absence of long-term follow-up restricts assessment of sustained recovery. To strengthen the evidence base, there is a pressing need for multicenter trauma registries and prospective investigations across sub-Saharan Africa. Such collaborative efforts would allow more accurate benchmarking, help identify context-specific prognostic factors, and guide the development of standardized protocols to improve outcomes in resource-limited environments.
